# Effects of integrating a structured design thinking strategy into generative AI-supported design learning on students’ design achievement, creative self-efficacy, and problem-solving skills

**DOI:** 10.3389/fpsyg.2026.1847432

**Published:** 2026-06-17

**Authors:** Fenglin Song, Biao Xu

**Affiliations:** Centre for Instructional Technology and Multimedia, Universiti Sains Malaysia, Penang, Malaysia

**Keywords:** creative self-efficacy, design education, design thinking, generative artificial intelligence, problem-solving skills

## Abstract

The rapid development of generative artificial intelligence (GAI) offers new opportunities for design education, yet its educational value may be limited without clear instructional guidance. To address this issue, this study integrates a structured design thinking strategy based on the Double Diamond model into GAI-supported design learning to support students’ purposeful and reflective use of GAI tools. An experimental study was conducted in a university-level character design course in China, involving 120 undergraduate students assigned to three conditions: GAI-supported learning with a structured design thinking strategy (GAI-SDTS), GAI-supported learning without the strategy, and traditional design learning. Results showed that students in the GAI-SDTS group achieved higher design achievement, creative self-efficacy, and problem-solving skills than those in the other two groups. These findings suggest that while GAI can support design learning, its educational benefits appear to be strengthened when embedded within a structured design thinking framework. The study also discusses the theoretical and practical implications of integrating generative AI with structured design thinking in higher education design contexts.

## 1 Introduction

Design education aims to cultivate students’ abilities in creativity, visual expression, and problem-solving (Calavia et al., 2021; Chiu and Hwang, 2025; Lin and Chang, 2024). In university-level design courses, the goal is not only to develop technical skills but also to help students form original ideas and apply them to real-world contexts. Traditional design teaching often relies on instructor-led demonstrations and tool-based practice, with students learning software such as Adobe Photoshop or Illustrator through repeated technical exercises (Hu and Li, 2025). While this approach provides foundational skills, it can also present challenges. Many students with creative ideas may find it difficult to express those ideas due to their limited technical proficiency. In some cases, the tools themselves become a barrier, restricting both the fluency of design expression and the confidence to explore alternative solutions (Rong et al., 2025). This gap between idea generation and execution may prevent students from fully realizing their design potential.

In recent years, the rapid development of generative artificial intelligence (GAI) has opened new possibilities for design education (Chandrasekera et al., 2025; Hwang and Chen, 2023; Petrova and Kuhnen, 2025). Tools such as ChatGPT, Midjourney, and Stable Diffusion allow students to generate images, explore visual styles, and receive text-based feedback using simple prompts (Derevyanko and Zalevska, 2023). These tools can help students express abstract ideas more quickly, reduce the time needed for trial and error, and support visual exploration even when technical drawing skills are limited (Rong et al., 2025; Yao et al., 2025). GAI is increasingly viewed not just as a production tool but also as a learning assistant that supports idea development and creative thinking (Hwang and Chen, 2023). Several studies have shown that integrating GAI into design activities can increase student engagement, improve creative outcomes, and reduce cognitive load during complex design tasks (Bian et al., 2025; Montenegro, 2024; Zeng et al., 2025). These advantages suggest that GAI has the potential to enhance the learning process in design classrooms. However, the way it is used can strongly influence its actual effectiveness.

Although GAI offers valuable support in design education, relying on it without clear instructional guidance can lead to several problems (Huang, 2024). When students engage with GAI tools independently, they may focus too heavily on output quality while neglecting the underlying thinking process (Saritepeci and Yildiz Durak, 2024). The ease of generating content may reduce their motivation to explore problems deeply or evaluate solutions critically (Melisa et al., 2025). For example, students may accept AI-generated results without independent thinking (Darvishi et al., 2024), which limits their ability to identify user needs, justify design decisions, or revise based on feedback. Without a structured learning framework, the use of GAI can weaken students’ design reasoning and reduce opportunities for meaningful learning. These challenges highlight the need for a pedagogical strategy that not only integrates GAI into the classroom but also supports students in using it thoughtfully and purposefully (Caires et al., 2023).

Design thinking (DT) is a human-centered approach to innovation that emphasizes empathy, creativity, and iterative problem solving (Razzouk and Shute, 2012; Ramos et al., 2024). In the context of design education, DT helps students better understand user needs, define problems clearly, explore multiple ideas, and test solutions through feedback and refinement (Lin et al., 2025). It encourages learners to move beyond surface-level aesthetics and engage in deeper reasoning about function, context, and user experience. As a teaching approach, DT aligns well with the goals of design education by promoting active inquiry, collaboration, and reflection. Prior studies have shown that DT-based instruction can enhance students’ creative confidence (Balakrishnan, 2022), increase their engagement in open-ended tasks (Saad et al., 2020), and improve their ability to solve complex and real-world problems (Tsai et al., 2023). While the value of DT is widely recognized, its effective implementation in classroom settings requires structured strategies that can guide students through each stage of the design process (Wei et al., 2025). One way to transform DT into an effective classroom strategy is to provide students with a clear structure that guides their process from problem identification to solution development.

The Double Diamond model, developed by the UK Design Council (2007), is one such approach that has been widely applied in both professional and educational design contexts. It consists of four key stages: Discover, Define, Develop, and Deliver. These stages help students separate divergent and convergent thinking, understand the problem space thoroughly, and generate user-centered solutions through iteration and feedback (Chang and Tsai, 2024; Yang and Lin, 2025). In instructional settings, the Double Diamond model provides a practical framework that helps students internalize DT as a repeatable, reflective process rather than a one-time activity (Saad et al., 2020; Yang and Lin, 2025). However, few studies have examined how the Double Diamond model can be integrated with GAI tools to support design learning in higher education settings (Grau and Rockett, 2026).

In this study, the Double Diamond model is used as the DT strategy aimed at guiding students in their use of GAI tools. This structured approach is expected to help students engage more meaningfully with their design tasks, make better use of AI-generated resources throughout the design process. To evaluate the effectiveness of this structured DT strategy supported by GAI tools, an experimental study was conducted in a university-level character design course focused on card game character design in China. Based on this instructional context, the following research questions were formulated to guide the investigation:

RQ1: Does the Generative AI-supported learning with a Structured Design Thinking Strategy (GAI-SDTS) approach lead to significantly higher design achievement compared to Generative AI-supported learning without the strategy (GAI) and Traditional Design Learning (TDL)?

RQ2: Does the GAI-SDTS approach enhance students’ creative self-efficacy compared to the GAI and TDL groups?

RQ3: Do students in the GAI-SDTS group demonstrate stronger problem-solving skills than those in the GAI and TDL groups?

To complement these research questions, the study further proposed the following hypotheses:

RH1: Students in the GAI-SDTS group would achieve higher design achievement than those in the GAI and TDL groups.

RH2: Students in the GAI-SDTS group would report higher creative self-efficacy than those in the GAI and TDL groups.

RH3: Students in the GAI-SDTS group would demonstrate stronger problem-solving skills than those in the GAI and TDL groups.

## 2 Literature review

### 2.1 GAI in design education

Since the release of GAI tools such as ChatGPT, Midjourney, and Stable Diffusion, their applications in design education have rapidly expanded (Li et al., 2025). In learning activities, GAI can assume multiple educational roles, such as teacher, student, learning peer, domain expert, administrator, and learning tool (Hwang and Chen, 2023), which support learners in a wide range of design activities. Multiple studies have examined GAI in a range of design education contexts (Almaz et al., 2024; Chandrasekera et al., 2025; Chen et al., 2025; Petrova and Kuhnen, 2025). For example, Chandrasekera et al. (2025) examined the use of GAI tools in relation to diverse learning styles, while Almaz et al. (2024) discussed the integration of GAI into architectural and interior design education. Petrova and Kuhnen (2025) further investigated the role of GAI as a conceptualization and research tool in design, and Chen et al. (2025) examined the use of a ChatGPT-driven pedagogical agent in design and art courses. However, existing studies have primarily employed GAI directly while overlooking the role of instructional support. Therefore, this study proposes that integrating GAI into DT may be beneficial for enhancing students’ interaction with GAI and improving their performance in design activities.

### 2.2 Design thinking

DT was first introduced by Rowe (1991) and has been described as the cognitive process of designers (Balakrishnan, 2022; Dunne and Martin, 2006). Scholars regard DT as a constructive educational approach because it transforms students’ learning processes by linking real-world experiences, problem-solving foundations, and cycles of reflection and reconstruction (Tsai et al., 2023; Tu et al., 2018). As a thinking framework, DT is also considered an effective means of cultivating key twenty-first century skills such as creativity, critical thinking, collaboration, and problem-solving (Razali et al., 2022; Du et al., 2012; Guaman-Quintanilla et al., 2022).

With the development of DT across different fields, various DT models have emerged. The DT model proposed by the Hasso-Plattner Institute of Design at Stanford University involves five key stages, which consist of Empathy, Definition, Ideas, Prototype, and Testing (Hasso Plattner Institute of Design at Stanford University (d.school), 2010). The UK Design Council (2007) introduced a four-stage DT process, known as the “Double Diamond,” comprising the phases of Discover, Define, Develop, and Deliver. Each phase contains iterative loops through which ideas can be explored and tested. IDEO, a design innovation consultancy, also proposed a human-centered DT model that includes three steps, which are inspiring solutions, ideating solutions, and implementing solutions (Liu et al., 2024). Based on the Danish Design Ladder, Wrigley and Straker (2015) proposed an Educational Design Ladder that illustrates pedagogical stages in the development of DT. This model includes five phases, which are foundation level, product level, project level, business level and professional level.

Among these models, the Double Diamond model has become one of the most structured and widely applied frameworks in design education (Lai et al., 2023; Li et al., 2025; Yang and Lin, 2025). For example, Yang and Lin (2025) incorporated the Double Diamond model into a design curriculum to cultivate students’ abilities in problem identification, solution construction, and expression feedback. This alignment makes the model particularly suitable for the present study, in which students were also required to progress through iterative phases of exploration, definition, development, and final presentation during character design. Therefore, the present study adopted the Double Diamond model as an design strategy to guide students’ design activities.

### 2.3 Creativity self-efficacy

Creativity is understood as the ability to draw upon prior knowledge to generate new ideas and develop innovative solutions (Kleiman, 2008; Liu et al., 2024). Creativity self-efficacy refers to individuals’ beliefs in their capacity to produce original ideas and engage in creative performance (Tierney and Farmer, 2002). Rooted in self-efficacy theory (Bandura, 1997; Schack, 1989), creativity self-efficacy emphasizes domain-specific confidence as a driver of motivation and creative engagement. Abulela (2024) argued that creativity self-efficacy is a critical attribute for predicting, developing, and enhancing creative performance. Creativity can also be assessed through individuals’ confidence in their creative abilities (Liu et al., 2024). Empirical studies indicate that students with higher levels of creativity self-efficacy maintain positive psychological states that support creative behavior (Lee et al., 2025). Moreover, compared with those with lower self-efficacy, individuals with stronger creative self-beliefs are more likely to experiment with alternative ideas and strategies when encountering task-related obstacles, thereby increasing their likelihood of success (Liu Z. et al., 2025; Paulus and Brown, 2007). Given its significance, it is crucial to examine how integrating a structured DT strategy into GAI-supported design learning affects the development of students’ creativity self-efficacy.

### 2.4 Problem-solving skills

Problem-solving is conceptualized as individuals’ perceptions of their competence when dealing with real-life personal problems, including confidence in problem-solving, approach-avoidance tendencies, and perceived personal control (Heppner and Petersen, 1982). Problem-solving and creativity are closely linked, as creative thinking facilitates the development of novel and effective solutions to complex challenges (Calavia et al., 2021). It involves translating abstract problems into concrete representations, generating potential abstract solutions, and converting those solutions into actionable strategies. Empirical evidence has shown that DT-based instructional approaches can effectively foster problem-solving skills (Liu et al., 2024; Tsai et al., 2023). For example, Liu et al. (2024) reported that applying the Stanford DT model significantly enhanced pre-service teachers’ inventive problem-solving skills. Similarly, Tsai et al. (2023) characterized DT as a student-centered approach that enhances students’ wicked problem-solving capabilities. In addition, recent studies suggest that GAI can contribute to the development of problem-solving skills. For example, Kim et al. (2025) found that GAI can facilitate structured problem-solving, whereas Wei et al. (2025) demonstrated that integrating GAI tools into digital activities can significantly enhance students’ collaborative problem-solving skills. These findings suggest that integrating DT with GAI may provide an effective pedagogical structure for cultivating problem-solving skills. Therefore, this study examines how integrating a structured DT strategy into GAI-supported design learning influences students’ problem-solving skills.

## 3 Methods

### 3.1 Research design

**Figure 1:**
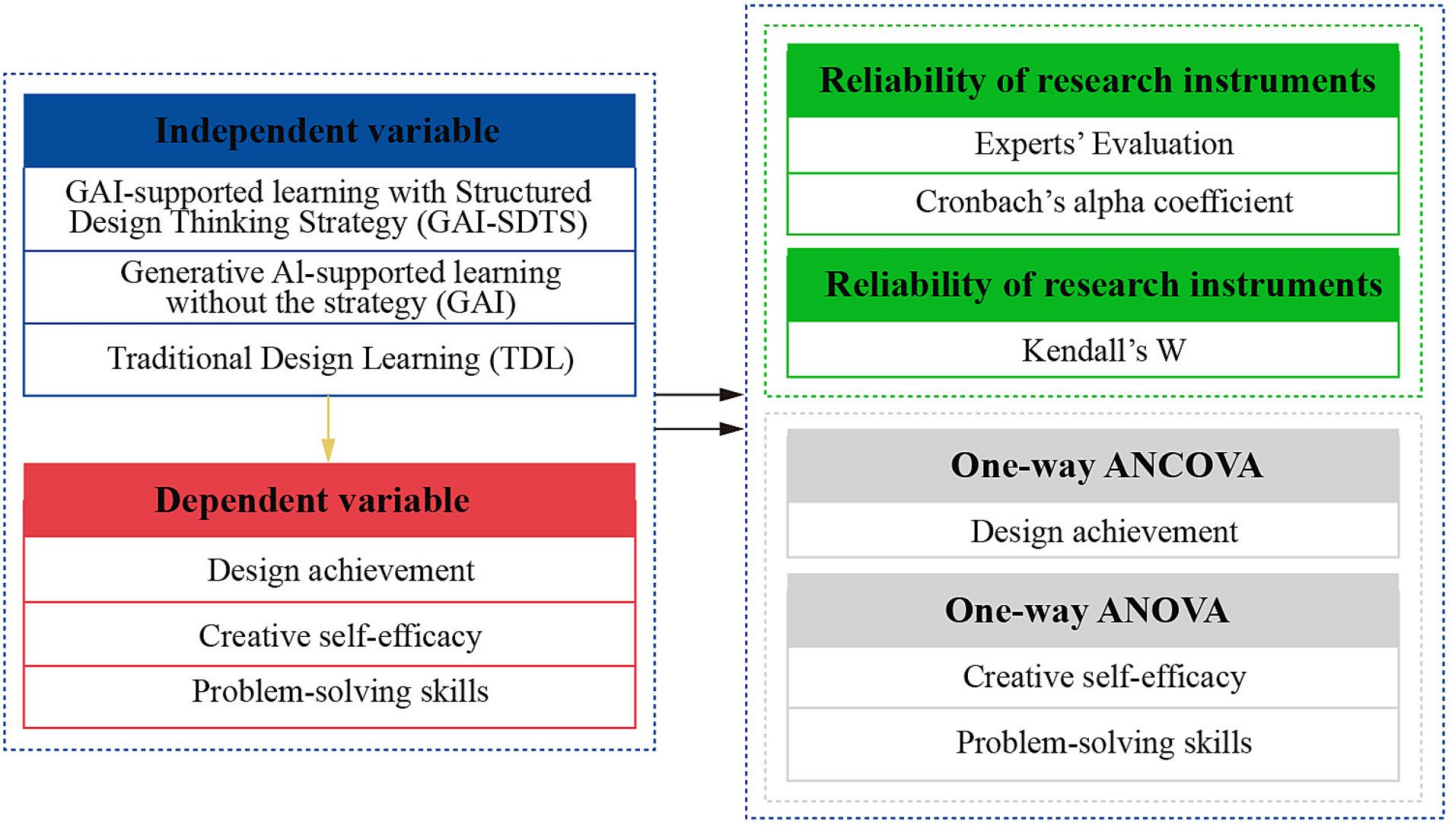
The research structure of the study.

This study adopted a quasi-experimental design that combined a posttest-only control group design for design achievement and a pretest-posttest control group design for creative self-efficacy and problem-solving skills. Quasi-experiments remain a rigorous approach for examining causal relationships in authentic educational settings where full experimental control is not feasible, provided that potential threats to validity are carefully addressed (Cook and Campbell, 1986; Chiu and Hwang, 2025; Liu et al., 2024). The independent variable was the instructional method used during the card game character design, with participants randomly assigned to one of three groups: (1) Generative AI-supported learning with Structured Design Thinking Strategy (GAI-SDTS), (2) Generative AI-supported learning without the strategy (GAI), and (3) Traditional Design Learning (TDL). The dependent variables were design achievement, creative self-efficacy, and problem-solving skills, as shown in Figure 1.

### 3.2 Population and sampling

The population for this study consisted of second-year undergraduate students majoring in animation at a university located in central China. A total of 120 students aged between 19 and 22 years (*M* = 20.4, SD = 0.84) participated in the experiment. A single-stage cluster sampling method was used to select three classes out of six existing animation classes. All students in the selected classes were then randomly assigned to one of three groups:

1. Generative AI-supported learning with the Structured Design Thinking Strategy (GAI-SDTS), in which students received guidance based on the Double Diamond DT framework while using generative AI tools;
2. Generative AI-supported learning without the strategy (GAI), where students used GAI tools but without any structured design guidance; and.
3. Traditional Design Learning (TDL), which followed a conventional design instruction model without GAI tools or explicit DT strategies.

A four-week intensive practical course on character design was conducted from October 20, 2025, to November 14, 2025. Students were reorganized into new mixed-class groups based on their assigned condition and completed the card character design tasks independently. Meanwhile, written informed consent was obtained from all participants prior to the study. To ensure instructional consistency, the instructor introduced the same course schedule, design requirements, and learning objectives across all groups. During the design process, they avoided intervening in students’ creative work and focused only on maintaining classroom order, which included time management, monitoring students’ adherence to task requirements, and responding to procedural questions. This combination of cluster sampling and random group assignment ensured the initial equivalence of participants across groups and helped address the lack of a pretest measure for design achievement, thus enhancing the internal validity of the experimental design.

### 3.3 Course design

**Figure 2:**
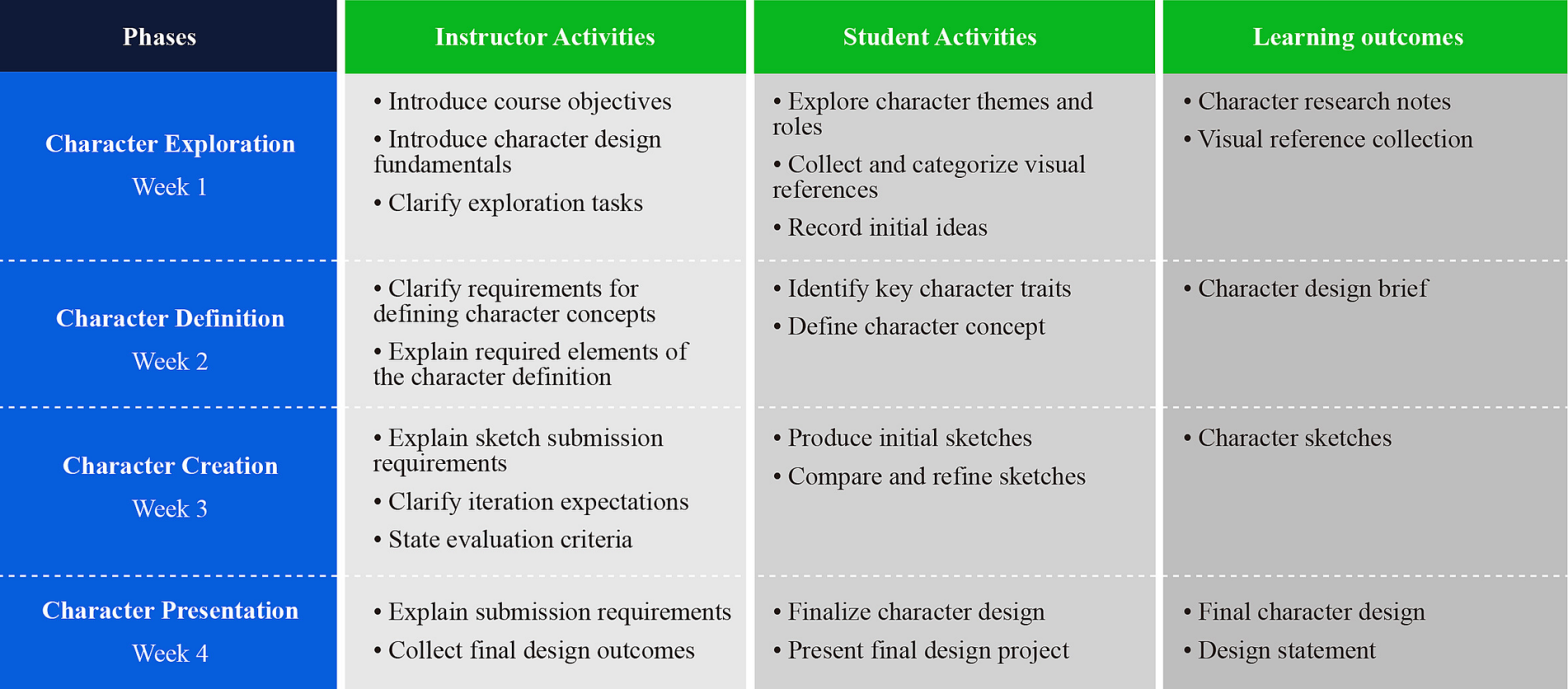
Course design.

A course titled “Card Game Character Design” was specifically developed for this study to enable students to effectively integrate digital technologies into their design practice. The objective of the practical course was as follows: “Using the digital tools you have learned, design and create a distinctive card game character with clear personality traits.” The course structure comprised four key components: (a) character exploration, (b) character defining, (c) character creation, and (d) character presentation, as shown in Figure 2. These components were designed to engage students in creatively addressing both theoretical and practical design problems.

To minimize instructional variability, the instructional design strictly controlled all variables across groups except for the presence of the structured DT strategy. All groups shared identical learning objectives, design tasks, time allocation, instructor, instructional materials, and assessment criteria. The instructor had over 15 years of experience in design education and more than 2 years of experience using GAI tools in instructional contexts. In addition, the instructor received prior training to standardize instructional delivery across all conditions, with an emphasis on neutral facilitation and minimizing variability unrelated to the experimental intervention.

Students in the TDL group conducted design exploration primarily through printed materials and online resources and independently formulated character information based on the collected references. They then created hand-drawn sketches and completed their final designs using traditional digital tools, such as Photoshop. In contrast, students in both the GAI-SDTS and GAI groups had access to the same GAI tools, Deepseek and Dreamina, to support their design processes. Deepseek facilitated text-based interaction, enabling students to develop character-related information through real-time dialogue and iterative refinement. Dreamina supported text-to-image and image-to-image generation, allowing students to produce and refine high-quality character visuals by adjusting parameters and prompts.

However, the main difference between the two GAI-supported groups lay in whether the Double Diamond DT framework was explicitly operationalized. Specifically, students in the GAI-SDTS group were provided with a Double Diamond-Based Design Operation Sheet (DDDOS) that articulated stage objectives, self-monitoring questions, suggested use of GAI tools, and stage completion check. Details of the DDDOS are provided in Supplementary Table S1. This sheet supported students’ independent monitoring of their design progress without additional instructor intervention. In contrast, students in the GAI group used the same GAI tools freely without structured design guidance.

**Figure 3:**
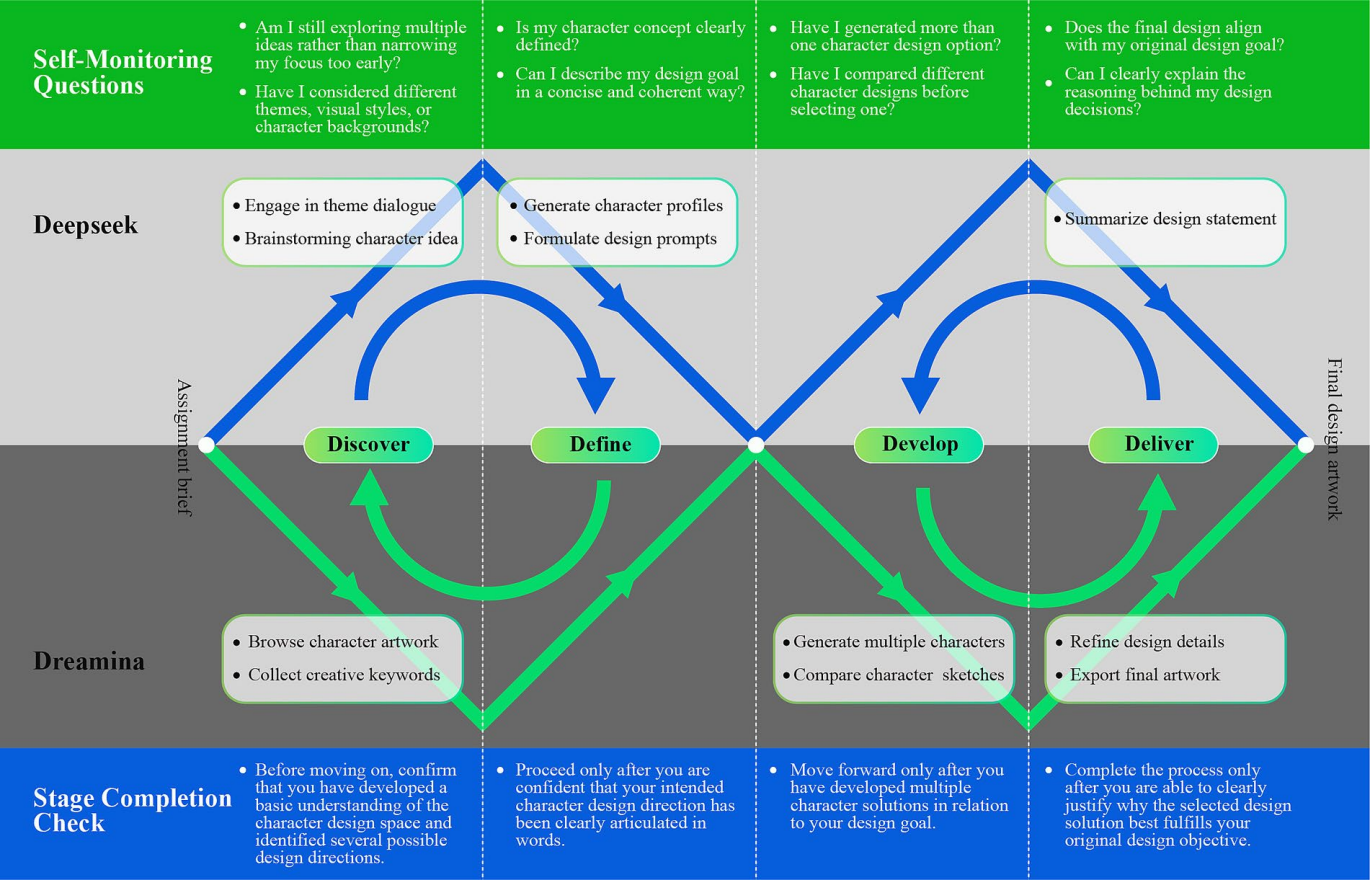
GAI-supported learning with the SDTS.

The DDDOS for the GAI-SDTS group was structured around the four stages of the Double Diamond model: (1) Discover, (2) Define, (3) Develop, and (4) Deliver. Throughout the DDDOS, GAI-SDTS group were encouraged to use GAI tools at designated points aligned with each design stage to support creativity and problem-solving. Rather than providing instructional guidance or evaluative feedback, the sheet externalized the DT, enabling students to independently regulate their progression through the design task while maintaining instructional equivalence across experimental conditions. Although the stages were presented sequentially, the design process was inherently iterative; students were encouraged to revisit earlier stages whenever reflection revealed insufficient exploration, unclear definition, or misalignment between design intent and outcomes. Figure 3 illustrates the GAI-supported learning with the structured DT strategy for the GAI-SDTS group.

**Figure 4:**
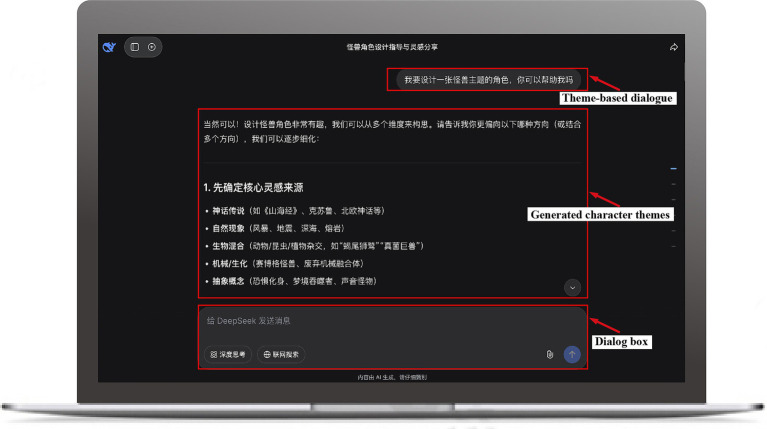
Exploring character themes through dialogue with Deepseek.

**Figure 5:**
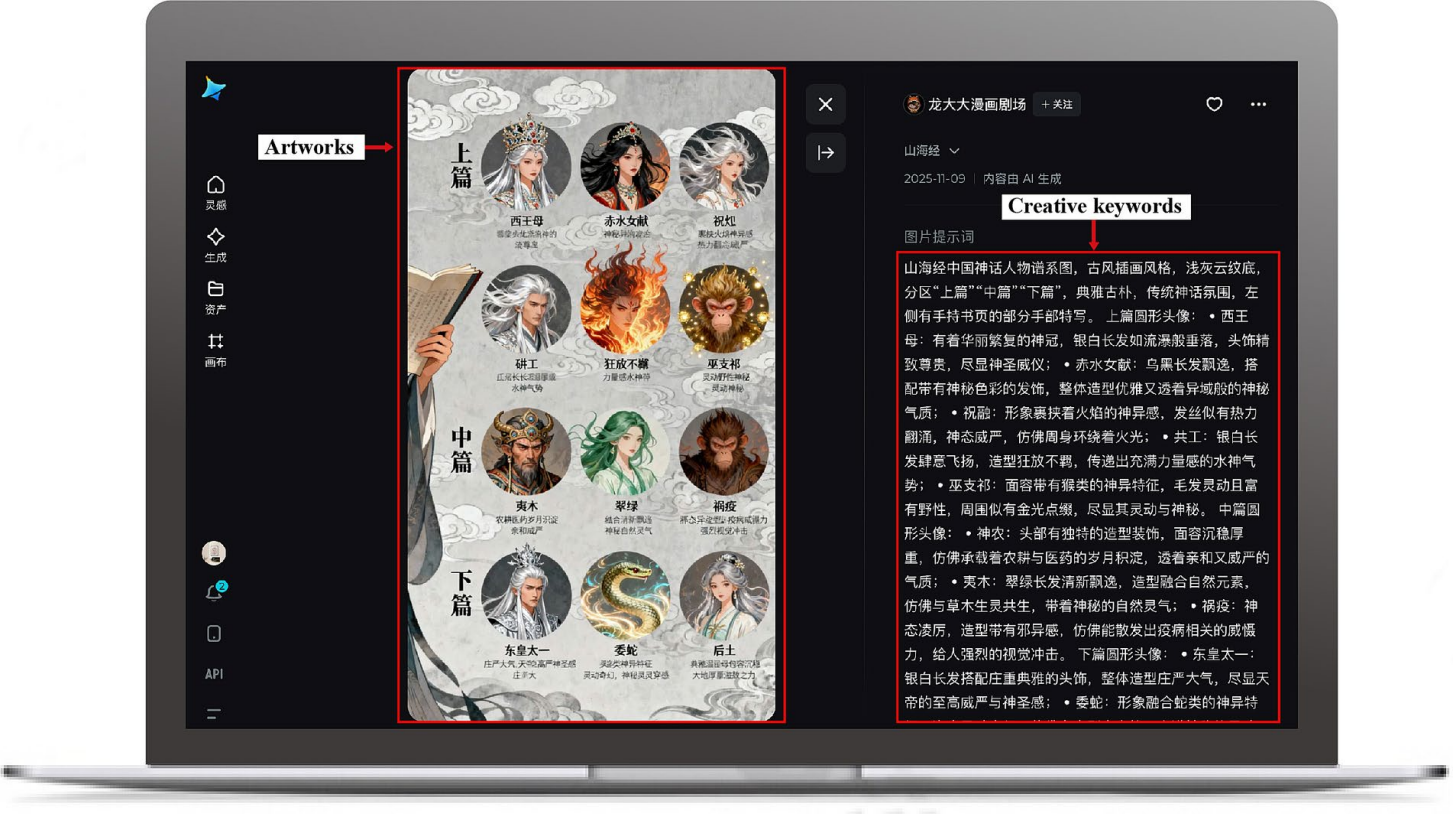
Browsing artworks and collecting keywords using Dreamina.

In the Discover phase, the DDDOS guided GAI-SDTS group to engage in broad design exploration by exploring diverse themes, contexts, and visual possibilities for character design. Students were prompted to reflect on whether they were sufficiently exploring multiple ideas rather than prematurely narrowing their focus. At this stage, DeepSeek was used to engage students in theme-based dialogue to support the brainstorming of character ideas, as illustrated in Figure 4, while Dreamina was used to browse homepage artworks and collect creative keywords related to visual styles, as shown in Figure 5. Students proceeded to the next stage only after confirming that they had developed a basic understanding of the character design space and potential design directions.

During the Define phase, the DDDOS supported convergent thinking by requiring GAI-SDTS group to articulate a clear character concept to identify design direction. Self-monitoring questions prompted students to assess whether their character concept was clearly defined and whether their design goal could be expressed concisely and coherently. At this stage, Deepseek was used to generate and refine character concepts and to articulate prompt descriptions, whereas Dreamina was intentionally not used to avoid premature visual fixation. Students advanced to the next phase only after determining for themselves that the intended direction of their character design had been clearly articulated.

**Figure 6:**
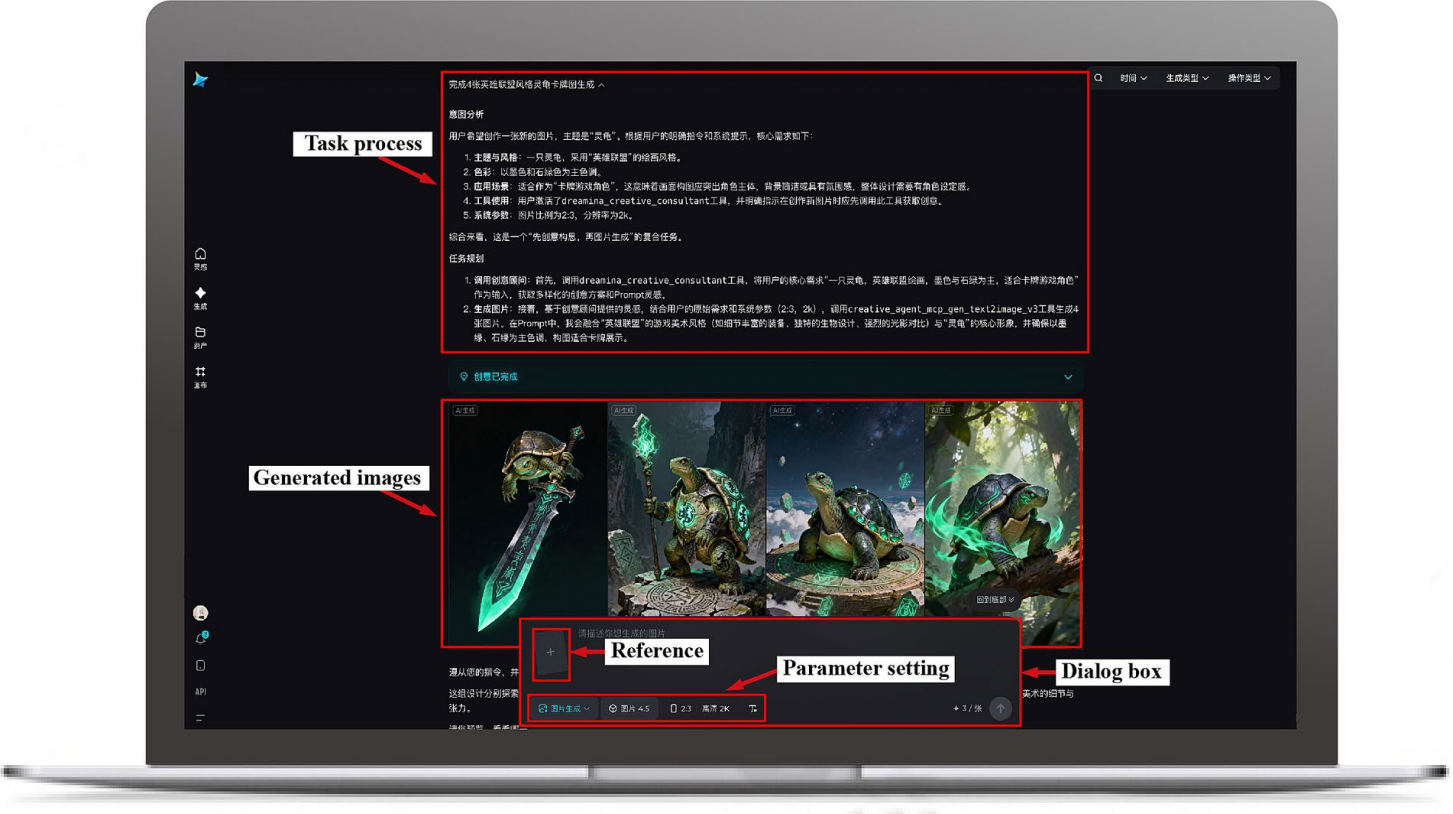
Generating multiple character variations based on predefined prompts using Dreamina.

In the Develop phase, the DDDOS guided GAI-SDTS group to generate, compare, and refine multiple character design solutions. Self-monitoring questions encouraged students to reflect on whether they had produced more than one design option and whether they had systematically compared alternatives before making selections. During this stage, Dreamina was used to generate multiple character variations based on predefined prompts and to support the comparison of alternative designs, as illustrated in Figure 6, while Deepseek was intentionally not used to allow students to focus exclusively on character sketch generation.

**Figure 7:**
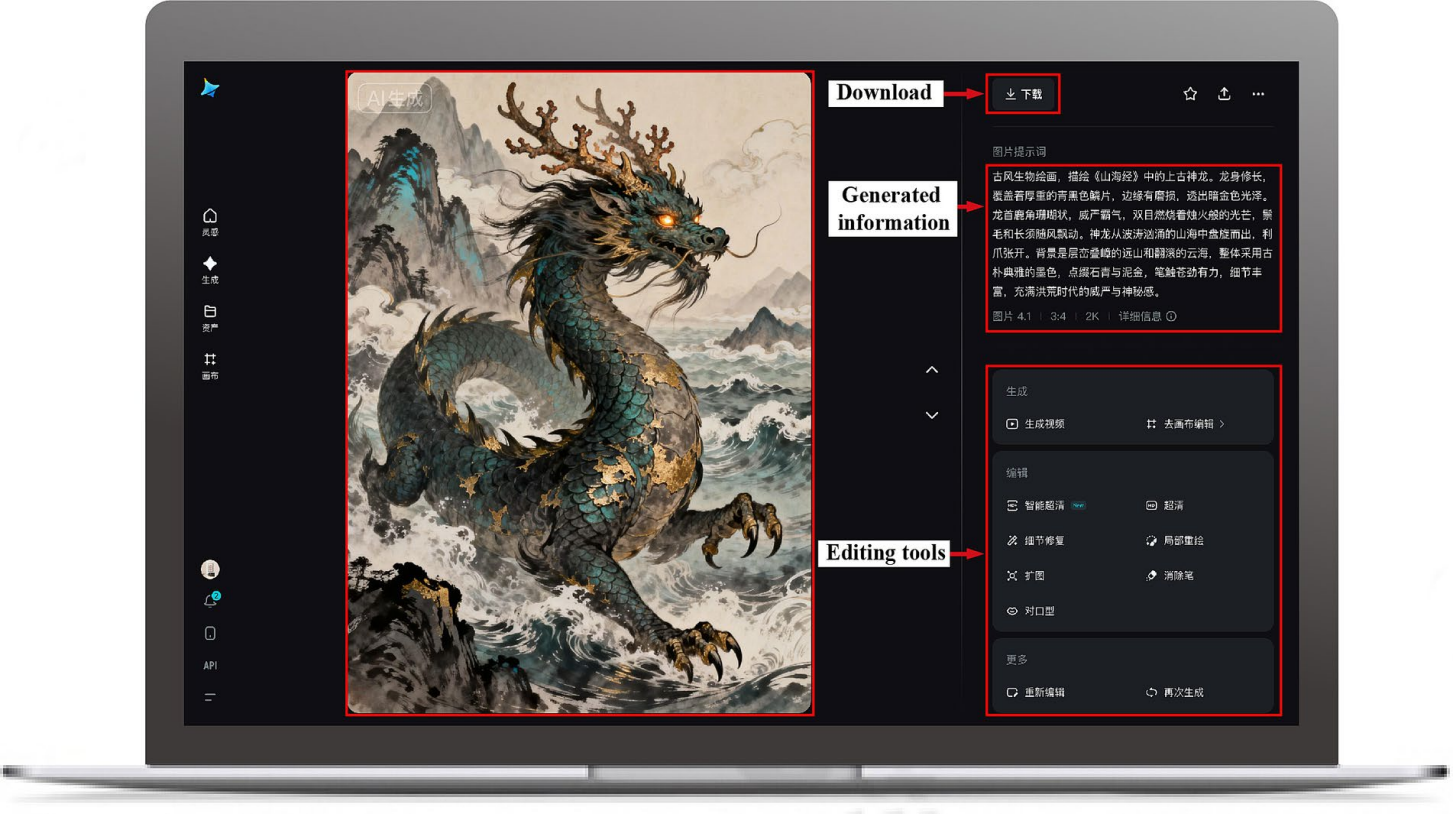
Refining visual details and exporting the final design using Dreamina.

During the Deliver phase, the DDDOS focused on finalization and presentation of the design outcome. It guided GAI-SDTS group to refine the selected character design and clearly articulate the rationale behind their design decisions. Self-monitoring questions prompted students to assess alignment between the final design and the original design goal and to evaluate their ability to justify design choices. At this stage, Dreamina was used to refine visual details and export the final design, as shown in Figure 7, while Deepseek was used to summarize the final design statement.

### 3.4 Instruments

The scoring rubric used to assess students’ design achievement was adapted from the framework proposed by Bian et al. (2025), originally developed for evaluating visual artwork in design education. To better align with the characteristics of the card-game character design task, the rubric was revised by two associate professors with over 11 years of teaching experience in design education. The modified rubric consists of five evaluation dimensions: (1) Technical Skill (2) Adherence to Theme (3) Composition and Design (4) Creativity (5) Detail Quality and Workload. Each dimension was rated on a five-point Likert scale, ranging from 1 (lowest) to 5 (highest). All student submissions were anonymized and randomly ordered before evaluation. Two instructors with 8 years of design teaching experience independently rated student’s final work. Inter-rater reliability was assessed using Kendall’s W, which yielded a value of 0.94 (*p* < 0.001), indicating a high degree of agreement between the two raters.

The Creative Self-Efficacy (CSE) scale, developed by Abulela (2024), was used to assess learners’ creative self-efficacy in relation to the GAI-integrated DT instructional approach adopted in this study. The CSE instrument consists of 21 items divided into two dimensions: Creative Ideas Self-Efficacy (CISE) with 12 items and Creative Performance Self-Efficacy (CPSE) with 9 items. All items were rated on a five-point Likert scale, ranging from 1 (strongly disagree) to 5 (strongly agree). To ensure the reliability of the instrument within the context of this study, internal consistency analyses were conducted. The overall Cronbach’s alpha coefficient for the CSE scale was 0.86, indicating a high level of reliability. The alpha value for the CISE dimension was 0.88, and for the CPSE dimension, it was 0.85, both demonstrating strong internal consistency.

The Problem-Solving Inventory (PSI), developed by Heppner and Petersen (1982), was employed to assess learners’ perceived problem-solving ability in the context of the GAI-integrated DT instructional approach implemented in this study. The PSI comprises 32 items distributed across three dimensions: Problem-Solving Confidence (PSC) with 11 items, Approach-Avoidance Style (AAS) with 16 items, and Personal Control (PC) with 5 items. All items were rated on a five-point Likert scale ranging from 1 (strongly disagree) to 5 (strongly agree). It is important to note that all three dimensions include reverse-scored items, where higher scores reflect more negative perceptions of one’s problem-solving ability. For example, a sample item from the PSC dimension is “Many problems I face are too complex for me to solve.” To examine the reliability of the instrument within the present study sample, internal consistency analysis was conducted. The overall Cronbach’s alpha coefficient was 0.86, indicating strong reliability. The alpha values for each subscale were 0.88 for PSC, 0.84 for AAS, and 0.82 for PC, suggesting good internal consistency across all dimensions. The PSI captures individuals’ confidence, approach tendencies, and perceived control in problem-solving, which remain relevant across domains, including design tasks. However, the PSI does not fully reflect design-specific and AI-mediated problem-solving processes. Therefore, the findings should be interpreted as general tendencies rather than domain-specific abilities.

### 3.5 Experimental procedures

**Figure 8:**
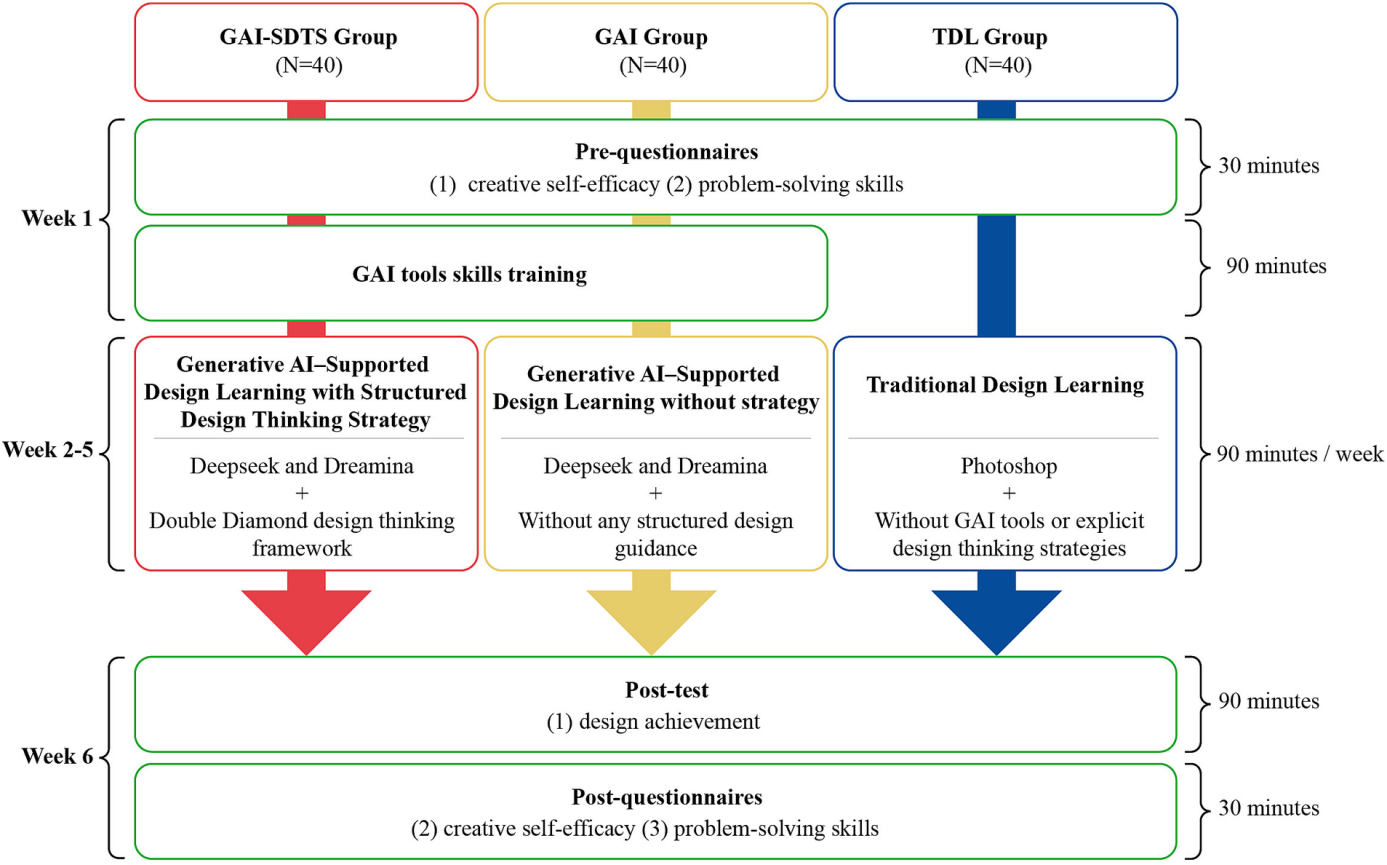
Experimental procedure.

The experimental procedure for this study is shown in Figure 8. In Week 1, all students completed the pre-tests of creative self-efficacy, and problem-solving skills. Students in the GAI-SDTS and GAI groups also received 90 min of standardized training on the use of GAI tools to ensure a shared understanding of how to apply these tools effectively during the design tasks. During the training, the instructor demonstrated the operational procedures of Deepseek and Dreamina, including prompt techniques, parameter adjustment,and image generation and refinement. Students also engaged in hands-on practice to familiarize themselves with the tools. This training was provided solely for operational familiarization and was deliberately kept independent of the experimental design content and tasks.

Between Weeks 2 and 5, students in all groups completed the same card game character design tasks. However, the instructional conditions differed across groups. The GAI-SDTS group completed the tasks using GAI tools guided by a structured DT strategy based on the Double Diamond model. The GAI group used the same GAI tools but without structured DT guidance. In contrast, the TDL group completed the tasks using traditional design software without the use of GAI tools or explicit DT strategies.

In the week 6 of the experiment, students’ final card character design artworks are used as the basis for grading their design achievements Meanwhile, all three groups completed the post-test questionnaires on creative self-efficacy and problem-solving skills.

## 4 Results

All data were analyzed by SPSS 26.0. As only post-test scores were collected for the design achievement, a one-way ANOVA was conducted to examine differences between the GAI-SDTS, GAI, and TDL groups. For the creative self-efficacy and problem-solving skills, both pre-test and post-test data were collected. Therefore, ANCOVA was used to assess post-test differences among groups while controlling students’ pre-test scores as covariates. The statistical significance level was set at *p* < 0.05 for all analyses.

### 4.1 Design achievement results

The assumption of homogeneity of variances was tested using Levene’s test, and the result was non-significant (*p* > 0.05), indicating that the variances were equal across groups and ANOVA assumptions were met. The ANOVA revealed a statistically significant effect of instructional condition on design achievement, *F* = 43.28, *p* < 0.001, suggesting that the type of instructional support had a substantial impact on students’ performance. As shown in Table 1, the GAI-SDTS group (*M* = 20.90, SD = 7.64) achieved significantly higher scores than both the GAI group (*M* = 18.65, SD = 7.41) and the TDL group (*M* = 16.13, SD = 6.85). *Post hoc* comparisons using Tukey’s HSD indicated that all pairwise differences were statistically significant: GAI-SDTS > GAI (*p* < 0.001), GAI-SDTS > TDL (*p* < 0.001), and GAI > TDL (*p* < 0.001). These results suggest that integrating a DT strategy into GAI-supported learning significantly enhanced students’ design achievement compared to both GAI-only and traditional learning.

**Table 1:** ANOVA result for design achievements of three groups.

Variable	Group	*N*	Mean	S.D.	*F*	*Post hoc*
Post-test	(1) GAI-SDTS	40	20.90	7.64	43.28***	(1) > (2)
	(2) GAI	40	18.65	7.41		(1) > (3)
	(3) TDL	40	16.13	6.85		(2) > (3)

The symbol *** indicates statistical significance at the *p* < 0.001 level. Note: *** *p* < 0.001.

### 4.2 Creative self-efficacy results

ANCOVA was conducted to examine the effects of instructional conditions on students’ creative self-efficacy, using pretest scores as covariates. The assumption of homogeneity of regression slopes was met, with *F* = 0.12, *p* = 0.89 > 0.05, indicating no significant interaction between the covariate and the grouping variable. Levene’s test of equality of error variances also confirmed that the data satisfied the assumption of homogeneity (*p* > 0.05). As shown in Table 2, the ANCOVA revealed a statistically significant main effect of group on creative self-efficacy, with *F* = 803.16, *p* < 0.001. After adjusting for pre-test scores, the GAI-SDTS group (adjusted *M* = 4.25) performed significantly better than both the GAI group (adjusted *M* = 3.84) and the TDL group (adjusted *M* = 3.54). Bonferroni *post hoc* analysis indicated that all pairwise group differences were statistically significant: GAI-SDTS scored higher than GAI, and GAI scored higher than TDL. These findings indicate that the GAI-SDTS group demonstrated the highest overall creative self-efficacy among the three groups.

**Table 2:** ANCOVA result for creative self-efficacy of three groups.

Variable	Group	*N*	Mean	S.D.	Adjusted mean	*F*	*Post hoc*
Total	GAI-SDTS	40	4.24	0.15	4.25	803.16***	(1) > (2)
	GAI	40	3.84	0.14	3.84		(1) > (3)
	TDL	40	3.55	0.14	3.54		(2) > (3)
Creative ideas self-efficacy	GAI-SDTS	40	4.26	0.19	4.26	432.90***	(1) > (2)
	GAI	40	3.81	0.16	3.80		(1) > (3)
	TDL	40	3.45	0.19	3.45		(2) > (3)
Creative performance self-efficacy	GAI-SDTS	40	4.22	0.19	4.23	190.10***	(1) > (2)
	GAI	40	3.89	0.14	3.89		(1) > (3)
	TDL	40	3.68	0.16	3.67		(2) > (3)

The symbol *** indicates statistical significance at the *p* < 0.001 level. Note: *** *p* < 0.001.

To further examine the dimensions of creative self-efficacy, ANCOVA was separately conducted on the creative ideas self-efficacy dimension. The test for homogeneity of regression slopes showed no significant violation, with *F* = 0.61, *p* = 0.55 > 0.05, and Levene’s test confirmed the equality of variances (*p* > 0.05). Results showed a significant group effect, with *F* = 432.90, *p* < 0.001. The adjusted means indicated that the GAI-SDTS group (4.26) reported the highest level of creative ideas self-efficacy, followed by the GAI group (3.80) and then the TDL group (3.45). All between-group differences were statistically significant. These findings indicate that the GAI-SDTS group achieved the highest level of creative ideas self-efficacy.

A similar pattern was observed in the creative performance self-efficacy dimension. The assumption of homogeneity of regression slopes was met, *F* = 0.38, *p* = 0.69 > 0.05, and Levene’s test showed no violation of the homogeneity of variances assumption (*p* > 0.05). ANCOVA results demonstrated a significant difference among the three groups, *F* = 190.10, *p* < 0.001. The adjusted post-test mean of the GAI-SDTS group (4.23) was significantly higher than that of the GAI group (3.89), which in turn was higher than the TDL group (3.67), with all differences reaching statistical significance. It is evident that the GAI-SDTS group reported the highest creative performance self-efficacy.

### 4.3 Problem-solving skills results

To evaluate the effects of instructional conditions on students’ problem-solving skills, a one-way ANCOVA was performed using pretest scores as covariates. The assumption of homogeneity of regression slopes was satisfied (*F* = 0.31, *p* = 0.72 > 0.05), and Levene’s test also indicated equality of error variances (*p* > 0.05). The analysis revealed a significant main effect of group on overall problem-solving skills, *F* = 461.14, *p* < 0.001. After adjusting for pre-test scores, the GAI-SDTS group (adjusted *M* = 4.44) scored higher than the GAI group (adjusted *M* = 4.04), and both outperformed the TDL group (adjusted *M* = 3.71), as shown in Table 3. These results suggest that students in the GAI-SDTS group reported relatively higher overall problem-solving skills.

**Table 3:** ANCOVA result for problem-solving skills of three groups.

Variable	Group	*N*	Mean	S.D.	Adjusted Mean	*F*	*Post hoc*
Total	GAI-SDTS	40	4.45	0.12	4.44	461.14***	(1) > (2)
	GAI	40	4.04	0.12	4.04		(1) > (3)
	TDL	40	3.72	0.10	3.71		(2) > (3)
Problem-solving confidence	GAI-SDTS	40	4.73	0.20	4.73	140.48***	(1) > (2)
	GAI	40	4.39	0.24	4.41		(1) > (3)
	TDL	40	4.00	0.15	4.00		(2) > (3)
Approach avoidance style	GAI-SDTS	40	4.34	0.11	4.34	228.83***	(1) > (2)
	GAI	40	4.03	0.10	4.03		(1) > (3)
	TDL	40	3.83	0.14	3.82		(2) > (3)
Personal control	GAI-SDTS	40	4.15	0.36	4.11	171.68***	(1) > (2)
	GAI	40	3.25	0.49	3.29		(1) > (3)
	TDL	40	2.75	0.30	2.75		(2) > (3)

The symbol *** indicates statistical significance at the *p* < 0.001 level. Note: *** *p* < 0.001.

For the problem-solving confidence dimension, the homogeneity of regression slopes was confirmed (*F* = 0.51, *p* = 0.61 > 0.05), and Levene’s test also showed no violation of variance equality (*p* > 0.05). The ANCOVA yielded a significant effect of group, *F* = 140.48, *p* < 0.001. The adjusted means revealed that the GAI-SDTS group (4.73) scored higher than the GAI group (4.01), and both groups scored higher than the TDL group (4.00), as shown in Table 3. These findings indicate that the GAI-SDTS group demonstrated the highest level of problem-solving confidence.

A similar pattern was found in the other two dimensions. For the approach-avoidance style, the regression slope assumption was satisfied (*F* = 0.67, *p* = 0.52 > 0.05), and Levene’s test supported homogeneity of variances (p > 0.05). The ANCOVA showed a significant group effect, *F* = 228.83, *p* < 0.001. The GAI-SDTS group (adjusted M = 4.34) outperformed the GAI group (4.03), which in turn scored higher than the TDL group (3.82). Regarding personal control, the assumption of equal regression slopes was met (*F* = 1.13, *p* = 0.33 > 0.05), with homogeneity of variances confirmed (p > 0.05). A significant difference was found among groups, *F* = 171.68, *p* < 0.001. The adjusted means were 4.11 (GAI-SDTS), 3.29 (GAI), and 2.75 (TDL), with all between-group differences reaching significance (see Table 3). These findings suggest that the GAI-SDTS group reported higher scores in both approach-avoidance style and personal control than the other groups.

## 5 Discussion

This study proposed a structured DT strategy to strengthen the use of GAI in design learning and to improve students’ design thinking ability. The strategy was implemented in a four-week intensive university-level character design course, and its effects were examined in terms of students’ design achievement, creative self-efficacy, and problem-solving skills.

### 5.1 Impact on design achievement

For the research question 1, the results indicate that students in the GAI-SDTS group achieved higher design scores than those in the GAI and TDL groups, while students in the GAI group also outperformed those in the TDL group. The findings indicate that the use of GAI can enhance students’ design achievement. This improvement appears to be associated with the affordances of GAI tools in expanding creative possibilities and supporting higher-quality design outcomes. Indeed, GAI can improve workflow, reduce cognitive load, and foster creativity in design learning (Chandrasekera et al., 2025), while also helping students explore design alternatives and support informed decision-making (Almaz et al., 2024). The results are consistent with the findings of Chen et al. (2025), who reported that a ChatGPT-driven pedagogical agent provided personalized and immediate feedback, significantly improving students’ academic performance in design and art courses. Similarly, Zeng et al. (2025) found that an AI-driven smart sketchpad offering greater creative freedom and expressive possibilities effectively enhanced students’ performance in film character design. However, it should be noted that students in the GAI-SDTS and GAI groups used AI tools to generate and refine visual outputs. Therefore, design achievement scores may partly reflect the quality of AI-generated artifacts rather than students’ independent design ability.

In addition, the findings suggest that integrating a structured DT strategy into GAI-supported learning environments can further improve students’ design achievement. This improvement may be attributed to the DDDOS, which provides learners with clear procedural guidance to plan, regulate, and reflect on their design processes. Meanwhile, the DDDOS offers explicit instructions on how to use GAI tools at different stages of the design process, which may help students engage with GAI in a more purposeful manner. In this way, the structured DT strategy appears to enhance the instructional effectiveness of GAI-supported design learning. This result supports earlier study that AI systems should be provided instructional scaffolding and possess the key attributes of a learning partner in order to strengthen the interaction between students and AI (Kim et al., 2022). It also aligns with Chiu and Hwang (2025), who demonstrated that integrating a mind-mapping strategy into GAI-supported learning environments significantly improved students’ learning achievement.

However, the present results differ from those reported by Zhan et al. (2022), who found no significant difference in learning achievement between product-based pedagogy and direct instruction in a high-school AI course. These differences may be attributed to the high degree of procedural complexity in Zhan et al.’s (2022) study. As product-based pedagogy organized learning around a seven-step 7P model (phenomenon, problem, plan, prototype, product, presentation, and price), the extensive process requirements may have fragmented students’ learning and diluted immediate performance gains. In contrast, the present study adopted a more streamlined and cognitively focused instructional structure. By embedding a structured DT strategy within GAI-supported learning, students were guided through essential phases of problem framing, ideation, and refinement without excessive procedural overhead.

### 5.2 Impact on creative self-efficacy

For the research question 2, students in the GAI-SDTS group reported higher creative self-efficacy than those in the GAI and TDL groups. This result can be attributed to the structured DT strategy embedded in the GAI-SDTS condition. Verganti et al. (2021) emphsied that DT can function as an instructional strategy that guides design activities through a set of practical principles, methods, and tools. In this study, the DT strategy functioned as an effective design approach that supported self-monitoring, encouraged task checking, and provided clear guidance on appropriate use of GAI, which prompted students to reflect on their design actions and make purposeful adjustments throughout the design process.

Meanwhile, GAI offered students opportunities to explore design ideas, clarify design goals, and generate and refine creative solutions. These factors contributed to more positive creative experiences, which strengthened students’ confidence in their creative abilities and supported a more fluent and sustained design process. This finding is consistent with the study by Chans et al. (2025), which reported that integrating GAI into DT activities can enhance students’ creativity by supporting idea generation and expanding the range of considerations involved in design. The results suggest that when GAI is embedded within a structured DT framework, it not only broadens the scope of creative exploration but also promotes reflective and user-centered design practices. Therefore, the integration appears to be particularly effective in strengthening students’ creative self-efficacy, as learners develop greater confidence in both generating creative ideas and transforming them into meaningful design outcomes.

With regard to creative ideas self-efficacy, the findings indicate that students in the GAI-SDTS group were more confident in their ability to generate original and meaningful ideas than students in the other two groups. This result is consistent with Petrova and Kuhnen (2025), who demonstrated that GAI effectively enhanced the ideation process and facilitated collaboration, which in turn fostered students’ confidence. Similarly, Chang and Tsai’s (2024) research showing that DT significantly and positively affected idea creativity in AI applications, particularly in terms of novelty and value. The combined use of a structured DT strategy and technological tools may further enhance this effect. However, Lively et al. (2023) cautioned that GAI may struggle to produce truly original ideas, as it relies heavily on existing works and patterns to generate design outputs. In addition, overreliance on AI may overshadow the innate creativity of humans and disconnect them from their creative instincts (Montenegro, 2024). The present study shows that integrating a structured DT strategy into GAI-supported learning can effectively address these concerns by guiding students to critically reflect on AI-generated ideas and to use GAI tools at designated points aligned with each stage of the design process.

A similar pattern was found for creative performance self-efficacy, which refers to students’ confidence in turning ideas into clear and well-developed design outcomes. Students in the GAI-SDTS group reported the highest creative performance self-efficacy. This finding is consistent with Lin and Chang’s (2024) study, which demonstrated that hands-on DT learning in AI-related tasks significantly enhanced the novelty, feasibility, and value of students’ design products. This finding suggests that creative self-efficacy during the execution stage depends strongly on structured guidance and opportunities for repeated improvement. Although GAI can improve visual quality and lower technical barriers, structured DT further helps students make informed design decisions. As a result, students develop stronger confidence in their ability to successfully realize creative ideas.

### 5.3 Impact on problem-solving skills

For the research question 3, the findings indicate that students in the GAI-SDTS group exhibited stronger overall problem-solving skills than those in the GAI and TDL groups, with consistent advantages observed across all three dimensions: problem-solving confidence, approach-avoidance style, and personal control. This may be because the structured DT functioned as a powerful pedagogical mechanism for cultivating problem-solving abilities in GAI-supported design learning environments. In the GAI-SDTS condition, learners maintained decision-making autonomy while benefiting from a clearly structured design process, which supported purposeful strategy use and sustained engagement, thereby contributing to improved problem-solving performance (Willems et al., 2025).

The results are consistent with Kim et al. (2025) study, which demonstrated that DT-based GAI improved problem-solving skills, suggesting that integrating DT with GAI enhances learners’ contextual understanding and emotional engagement, thereby supporting more effective problem definition. Similarly, Hong et al. (2023) found that DT-based GAI education led to significant improvements across multiple dimensions of creative problem-solving ability, including problem detection and analysis, idea generation, action planning, execution, and communication. These findings suggest that structured inquiry and iterative design cycles strengthen learners’ confidence and persistence in complex tasks.

However, Gonsalves (2024) cautioned that easy access to AI-generated solutions may encourage superficial engagement. In contrast, the present findings suggest that structured DT can effectively address common concerns about cognitive passivity or shallow learning associated with AI use. This indicate that the educational impact of GAI is largely shaped by pedagogical design. In the GAI-SDTS condition, learners were required to generate, compare, and justify alternative solutions, positioning AI outputs as prompts for reflection rather than ready-made answers.

With regard to problem-solving confidence, the higher confidence reported by the GAI-SDTS group may be attributed to the explicit emphasis on problem identification and definition during the early stages of the Double Diamond model. By guiding students to systematically explore design contexts and clarify constraints before generating solutions, the structured strategy likely reduced uncertainty. In contrast, students in less structured conditions may have relied more heavily on trial-and-error or AI outputs without fully understanding the underlying problem space, which can undermine confidence.

The improvements observed in approach-avoidance style further highlight the value of structured DT in GAI-supported learning. Students in the GAI-SDTS group were more inclined to actively engage with problems rather than avoid them, suggesting greater persistence and willingness to invest effort. In addition, gains in personal control suggest that students experienced stronger self-regulation and agency. The DDDOS externalized the design process without imposing direct instructor control, enabling students to monitor progress independently while maintaining autonomy. The dialogue records showed that students in the GAI-SDTS group were more willing to confront emerging design difficulties by iteratively refining prompts, requesting alternative perspectives, and testing multiple solution paths. These results show that integrating GAI into a structured DT framework enhances not only problem-solving outcomes but also learners’ confidence, engagement, and self-regulatory capacities in complex design tasks.

### 5.4 Contribution to recent research

In recent studies, GAI has been shown to improve learning outcomes in design education, including creativity, motivation, self-efficacy, cognitive load, and academic achievement (Chiu, 2026; Huang et al., 2026; Wang et al., 2026). Existing research has largely emphasized GAI itself, but has paid less attention to how integrating GAI with well-established DT instructional models shapes learning outcomes. In design education, DT is crucial, but research on how integrating GAI into DT influences students’ creative self-efficacy and problem-solving skills remains limited. The present study is the first to examine the differential effects of integrating a structured DT strategy into GAI-supported design learning for animation majors. The findings show that a structured DT strategy can function as a pedagogical mechanism that strengthens not only design achievement but also creative self-efficacy and problem-solving skills. The study therefore extends recent research by clarifying that the educational value of GAI depends not only on access to AI tools, but also on how its use is pedagogically structured across the design process.

### 5.5 Theoretical and practical implications

This study provides several important theoretical contributions to the understanding of GAI-supported design learning in higher education. Its novelty lies in the integration of DT, particularly the Double Diamond model, into GAI-supported design instruction. While previous studies have examined the role of GAI in design education, they have primarily treated AI as a single tool for content generation. In contrast, the present study conceptualizes GAI as a process-oriented learning support that is deliberately embedded across all stages of the DT cycle to guide students’ structured and purposeful use of AI. By integrating GAI with a structured DT strategy grounded in the Double Diamond model, this study extends prior research on GAI-supported creativity and problem-solving. In addition, this study contributes to design education theory by empirically validating the role of structured DT in enhancing design achievement, creative self-efficacy, and problem-solving skills within AI-mediated learning environments. Moreover, the results enrich theoretical discussions of human-AI collaboration by illustrating how guided interaction with AI can promote reflective thinking, self-regulation, and learner agency, rather than cognitive passivity.

From a practical perspective, this study offers actionable insights for educators, students, and institutions seeking to integrate GAI into design education. For educators, the findings highlight the importance of aligning GAI use with design stages and instructional goals, ensuring that students engage critically with AI-generated outputs rather than relying on them unreflectively. For students, the study suggests that scaffolded learning environments integrating GAI tools can support idea generation, refinement, and evaluation, while helping learners maintain coherence and cognitive focus throughout the design task. At the institutional level, the results suggest that effective adoption of GAI requires pedagogical frameworks and guidelines that mitigate overreliance and foster higher-order thinking.

### 5.6 Limitations and future directions

This study has several limitations that should be considered when interpreting the findings and that also suggest directions for future research. First, although the study provides evidence for the effectiveness of integrating a structured DT strategy into GAI-supported learning, it relied primarily on outcome-based measures and quantitative analyses. This is a limitation because DT has long been understood as a process-oriented form of learning (Scheer et al., 2012; Li et al., 2024). Future research would benefit from adopting qualitative or mixed-methods approaches to explore students’ learning experiences, design decision-making processes, and interactions with GAI tools across different stages of the design process. Second, this study compared the GAI-SDTS, GAI, and TDL groups, but did not include a structured DT strategy without GAI-supported learning group. Future studies could include an additional structured DT-only group to further clarify the independent and combined effects of structured DT and GAI-supported learning. Third, the PSI captures students’ general perceived problem-solving tendencies, which remain relevant across domains. Future research could incorporate design-specific measures to better capture students’ problem-solving processes in AI-supported design learning. Fourth, the instructional intervention in this study was limited to a specific design task and a single implementation of the Double Diamond-based DT strategy. This constrains transferability, as DT pedagogy has long been recognized as diverse in educational application (Hasso Plattner Institute of Design at Stanford University (d.school), 2010; Wrigley and Straker, 2015; Liu et al., 2024). Future studies could investigate how variations in DT structures or task complexity influence students’ performances. Fifth, this study did not examine long-term learning effects or potential risks. Cognitive offloading and overreliance on AI have been raised as challenges requiring further inquiry (Hewitt and Scardamalia, 1998; Wei et al., 2025). Longitudinal research is needed to investigate how sustained engagement with GAI within structured design frameworks affects students’ autonomy, critical engagement, and reflective thinking. Finally, this study did not incorporate instructor or peer critique into the intervention. This point is important because critique has long been regarded as a core pedagogical practice in design learning (Gray, 2013; Liu C. C. et al., 2025). While this strengthened internal validity, it also reduced the ecological authenticity of the learning environment. Future research should therefore examine whether the observed effects remain consistent when structured forms of critique are integrated across conditions.

## 6 Conclusion

This study examined the effects of integrating a structured DT strategy into GAI-supported design learning on university students’ design achievement, creative self-efficacy, and problem-solving skills. The research was guided by three hypotheses: students in the GAI-SDTS group would achieve higher design achievement (H1), report higher creative self-efficacy (H2), and demonstrate stronger problem-solving skills (H3) than those in the GAI and TDL groups. The results corroborated all three hypotheses. Across all measured outcomes, students in the GAI-supported learning with a structured DT strategy condition consistently outperformed those in the GAI-only and traditional design learning groups. These findings indicate that while GAI tools can effectively lower technical barriers and facilitate idea exploration, their educational value appears to be strengthened when embedded within a structured pedagogical framework. More importantly, the results suggest that the benefits of GAI in design education do not stem solely from content generation or efficiency gains, but from how AI use is pedagogically organized. By aligning GAI-supported activities with the stages of the Double Diamond model, the proposed approach supported students in systematic problem framing, iterative ideation, reflection, and refinement. The DDDOS externalized the design process without imposing direct instructor control, enabling learners to regulate their progress autonomously while maintaining engagement with complex design tasks.

## Data Availability

The raw data supporting the conclusions of this article will be made available by the authors, without undue reservation.
