# Supplementary material for: Effects of integrating a structured design thinking strategy into generative AI-supported design learning on students’ design achievement, creative self-efficacy, and problem-solving skills
Source: Front Psychol. 2026 Jun 17;17:1847432. doi: 10.3389/fpsyg.2026.1847432 (PMC13318989; doi:10.3389/fpsyg.2026.1847432)
Supplement: Supplementary file 1 [file Table_1.DOCX]

Supplementary Table S1

Double Diamond-Based Design Operation Sheet (DDDOS)

This Design Operation Sheet is designed to support your design process by applying the Double Diamond framework in combination with generative AI (GAI) tools. Rather than instructing you on what to design or evaluating your work, the sheet helps you structure your thinking, reflect on your progress, and make informed decisions about when and how to use GAI tools at each stage of the design process. You are required to complete each stage and conduct self-evaluation before proceeding to the next stage.

| **Stage 1：Discover (Character Exploration)** | |
| --- | --- |
| Objective | To support broad and divergent exploration of character design by examining multiple themes, narrative contexts, and visual possibilities, without prematurely fixing a design direction. |
| Self-Monitoring Questions | Am I still exploring multiple ideas rather than narrowing my focus too early?  Have I considered different themes, visual styles, or character backgrounds? |
| Potential Use of GAI Tools | Deepseek can support your early exploration by helping you: (1) engage in theme-based dialogue to expand possible story worlds and character roles;  (2) brainstorm multiple character ideas from different perspectives.  Dreamina can support visual exploration by allowing you to: (1) browse homepage artworks to observe a wide range of visual styles; (2) collect creative keywords related to form, mood, and aesthetic features.  At this stage, you should focus on exploration rather than producing finalized visuals. |
| Stage Completion Check | Before moving on, confirm that you have developed a basic understanding of the character design space and identified several possible design directions.  If your exploration feels too narrow or incomplete, revisit this stage to further expand themes, contexts, or visual references before proceeding. |
| **Stage 2：Define (Character Definition)** | |
| Objective | To converge on a specific design direction by articulating a clear and coherent character concept that could guide subsequent visual development. |
| Self-Monitoring Questions | Is my character concept clearly defined?  Can I describe my design goal in a concise and coherent way? |
| Potential Use of GAI Tools | Deepseek can support conceptual clarification by helping you:  (1) generate and refine character profiles;  (2) articulate structured prompt descriptions that translate abstract ideas into clear design intentions.  Dreamina is intentionally not used at this stage so that you can focus on conceptual clarity without being influenced by premature visual outcomes. |
| Stage Completion Check | Proceed only after you are confident that your intended character design direction has been clearly articulated in words.  If the concept remains vague or inconsistent, return to the Discover stage to refine your exploration before redefining the design direction. |
| **Stage 3：Develop (Character Design)** | |
| Objective | To generate, compare, and refine multiple character design solutions based on the predefined design concept. |
| Self-Monitoring Questions | Have I generated more than one character design option?  Have I compared different character designs before selecting one? |
| Potential Use of GAI Tools | Dreamina can support visual development by enabling you to:  (1) generate multiple character variations based on predefined prompts; (2) compare alternative visual solutions to inform your design choices.  Deepseek is intentionally not used at this stage so that you can concentrate on visual ideation, comparison, and refinement without further expanding the concept. |
| Stage Completion Check | Move forward only after you have developed multiple character solutions in relation to your design goal.  If the generated solutions do not adequately reflect the intended concept, revisit the Define stage to refine the design direction and prompts before further development. |
| **Stage 4：Deliver (Character Presentation)** | |
| Objective | To finalize the selected character design and clearly communicate both the final outcome and the rationale behind key design decisions. |
| Self-Monitoring Questions | Does the final design align with my original design goal?  Can I clearly explain the reasoning behind my design decisions? |
| Suggested Use of GAI Tools | Dreamina can support final production by allowing you to:  (1) refine visual details using editing tools;  (2) export the final character design.  Deepseek can support reflection and communication by helping you summarize and compose your final design statement. |
| Stage Completion Check | Complete the process only after you are able to clearly justify why the selected design solution best fulfills your original design objective.  If misalignment is identified, return to earlier stages to revise the concept or visual solutions before finalizing the design. |

***Note.*** This operation sheet functions as a self-monitoring tool rather than an instructional or evaluative instrument. It is designed to help learners track their design progress across stages independently, without additional teacher intervention. Although the stages are presented sequentially, the design process is inherently iterative. You are encouraged to revisit earlier stages whenever reflection reveals insufficient exploration, unclear definition, or misalignment between design intent and outcomes.
